# Comparison of the effects of probiotics, rifaximin, and lactulose in the treatment of minimal hepatic encephalopathy and gut microbiota

**DOI:** 10.3389/fmicb.2023.1091167

**Published:** 2023-03-24

**Authors:** Ming-Wei Wang, Wei-Juan Ma, Yan Wang, Xiao-Han Ma, Yu-Feng Xue, Jing Guan, Xi Chen

**Affiliations:** ^1^Department of Gastroenterology, The First Affiliated Hospital of Anhui Medical University, Hefei, Anhui, China; ^2^Anhui Provincial Key Laboratory of Digestive Disease, The First Affiliated Hospital of Anhui Medical University, Hefei, Anhui, China

**Keywords:** probiotics, rifaximin, lactulose, minimal hepatic encephalopathy, gut microbiota

## Abstract

**Background:**

Minimal hepatic encephalopathy (MHE) is an early stage in the pathogenesis of hepatic encephalopathy. Intestinal microbiota is involved in the pathogenesis of hepatic encephalopathy and has become an important therapeutic target. Since there is no unified treatment principle for MHE, this study was conducted to determine the safety and efficacy of different intestinal microecological modulators in the treatment of MHE, and to explore the potential mechanism through intestinal microbiota analysis.

**Methods:**

Patients with liver cirrhosis were screened for MHE using psychometric hepatic encephalopathy score test. Patients diagnosed with MHE were enrolled and received probiotics, rifaximin, or lactulose for 4 weeks. Adverse events were recorded. The psychometric hepatic encephalopathy score test was performed after treatment. Samples of blood and stool were collected at entry and 4 weeks. Blood samples were analyzed to assess blood ammonia, liver, kidney, and hemostatic functions. Stool microbiota were sequenced to confirm changes in microbial composition.

**Results:**

Of 323 patients with liver cirrhosis, 74 patients were diagnosed with MHE. In all, 54 patients were enrolled and 52 who agree to follow-up were included in analysis. The recovery rates of MHE patients received probiotics, rifaximin, and lactulose were 58.8% (20/34), 45.5% (5/11), and 57.1% (4/7), respectively. Probiotics and rifaximin improved liver function in MHE patients to a certain extent. Taxonomic compositions of gut microbiota in MHE patients were distinct from healthy people before treatment; the differences were significantly reduced after treatment, and the gut microbiota gradually resembled the structure of healthy individuals. We found that the relative abundance of specific taxa associated with anti-inflammatory and good cognitive functions was increased in MHE patients after treatment. Accordingly, metabolic pathways in MHE patients were altered before and after treatment. Downregulated pathways after probiotics treatment included glycometabolism and degradation of aromatic compounds. After lactulose treatment, degradation pathways of arginine and ornithine showed a downward trend.

**Conclusion:**

Probiotics, rifaximin, and lactulose are safe and effective in the treatment of MHE, and improve the composition of gut microbiota to some extent.

## Introduction

Hepatic encephalopathy (HE) is a common and serious complication of both chronic liver disease and acute liver failure. HE manifests as a wide spectrum of neuropsychiatric abnormalities, from subclinical changes (mild cognitive impairment) to serious disorientation, confusion, and coma (Rose et al., 2020). Minimal hepatic encephalopathy (MHE) is an insidious stage in the course of HE without obvious neuropsychiatric symptoms. It can only be diagnosed by a series of neuropsychological tests. Minimal or covert HE occurs in 20–80% of patients with cirrhosis, and MHE is associated with poorer health-related quality of life and motor vehicle driving accidents (Bajaj et al., 2009, 2020).

The pathogenesis of HE remains incompletely understood. Currently, ammonia poisoning remains the core theory, and the roles of inflammatory mediators and other toxic substances are also receiving increasing attention. Increasing blood ammonia can induce astrocyte swelling and cytotoxic brain edema, triggering neuropsychiatric symptoms. Systemic inflammatory state is increasingly considered a key factor to induce and aggravate HE. Direct evidence reveals elevated plasma inflammatory markers are associated with the occurrence and severity of HE, and synergy exists between elevated plasma inflammatory markers and high blood ammonia (Aldridge et al., 2015). Gut microbes have been a research hotspot in recent years, participating in many physiological processes of the host and playing important roles in the occurrence and development of many digestive diseases (Tilg et al., 2016). A previous study has reported that intestinal microbiota changes in patients with decompensated cirrhosis are characterized by a relative decrease of potentially beneficial autochthonous taxa, particularly Lachnospiraceae, Ruminococcaceae, and Clostridiales XIV, with relative overgrowth of potentially pathogenic taxa, e.g., Staphylococcaceae, Enterobacteriaceae, and Enterococcaceae (Bajaj et al., 2014b). They also found that the relative abundance of Lactobacillaceae in stool and salivary microbiota was higher in cirrhotic patients with MHE than in those without MHE; meanwhile, stool and salivary Lachnospiraceae genera (*Ruminococcus* and *Clostridium XIVb*) were associated with good cognition (Bajaj et al., 2019). Although no specific pathogen has been identified as the primary cause of MHE, several studies have revealed the important role of intestinal microbiota in cirrhosis and its complications. The concept of gut-liver-brain axis has also been increasingly mentioned. At present, drug intervention methods for MHE treatment by regulating intestinal microbiota mainly include lactulose, probiotics, and rifaximin (Patidar and Bajaj, 2015; Mancini et al., 2018). For example, lactulose treatment significantly improved the recovery rate of MHE, and gut microbiota changes in MHE patients modulate the effectiveness of this treatment (Wang et al., 2019). Probiotic intervention significantly reduced ammonia levels, small intestinal bacterial overgrowth, and orocecal transit time in cirrhosis, and improved psychometric hepatic encephalopathy score (PHES) (Lunia et al., 2014). Probiotic is safe and well-tolerated in cirrhosis and reduces endotoxemia and dysbiosis (Bajaj et al., 2014a). Rifaximin was associated with improved cognitive function and endotoxemia in MHE, which was accompanied by changes in the associations of gut bacteria with metabolites (Bajaj et al., 2013). In addition, studies have shown that combination drugs may be more effective than single agents (Sharma et al., 2013). A meta-analysis involving 25 studies has suggested that rifaximin followed by lactulose, probiotics plus L-Ornithine L-Aspartate (LOLA), and LOLA are most effective in reversing MHE (Dhiman et al., 2020). However, some studies have found no significant differences in efficacy among these drugs (Agrawal et al., 2012; Sidhu et al., 2016). The choice of MHE therapy remains controversial due to the time and dose of intervention, inconsistent inclusion criteria for patients, and the lack of direct efficacy comparison among different drugs. In addition, there is a lack of relevant studies on gut microbes in MHE patients.

The purpose of this study is to compare the safety and efficacy of different intervention drugs in the treatment of MHE. Combined with intestinal microbiota analysis in MHE patients before and after treatment, the potential mechanisms of MHE pathogenesis and the therapeutic effects of drugs are examined.

## Materials and methods

### Patients

This was a single-center trial and conducted at First Hospital of Anhui Medical University from December 2020 to March 2022. Inclusion criteria were: (1) 30–70 of age; (2) imaging demonstrating the presence of cirrhosis; (3) condition stabilized within 3 months; and (4) MHE was diagnosed using PHES. Patients were excluded if: (1) symptoms of gastrointestinal bleeding such as hematemesis and melena within the past 2 weeks or other serious complications of liver cirrhosis; (2) previous transjugular intrahepatic portosystemic shunting or shunt surgeries; (3) patients with a history of recent alcohol intake, recent infection, or antibiotic use (in the past 2 weeks); (4) patients on lactulose, LOLA, or probiotics therapy; (5) other serious diseases, including diabetes mellitus, heart failure, respiratory failure, end-stage renal disease, and neoplasms; and (6) long-term high-protein diet. The patient’s history and clinical characteristics were recorded at baseline.

### Ethics

The study was approved by the Hospital Ethics Committee of Anhui Medical University and registered in the department of Chinese Clinical Trial Registry (ChiCTR2000040960). All patients provided written informed consent. URL: http://www.chictr.org.cn/index.aspx.

### Diagnosis of MHE

All subjects were tested by the PHES system, including digital connection test five tests, i.e., number connection test A and B/C, digit symbol test, line tracing test, and serial dotting test. Stratified by age and education level, zero point meant that the difference values of MHE patients and control individuals was less than 1 standard deviation (SD). The final total score ranged from -15 to +5. According to the current internationally recognized diagnostic criteria for the PHES system, patients with total score of -4 or less were diagnosed with MHE, and MHE patients were considered as recovery while total score in the review exceeded -4.

### Treatment

Patients were assigned to the probiotics (Pro), rifaximin (Rif), or lactulose (Lac) groups for 4-weeks treatment. The Pro group received probiotics capsules (live combined *Bifidobacterium*, *Lactobacillus*, and *Enterococcus* Capsules, Oral) at 420 mg twice a day. The probiotics capsules containing *Bifidobacterium longum*, *Lactobacillus acidophilus*, and *Enterococcus faecalis* were produced by Shanghai Xinyi Pharmaceutical Co., Ltd. Each capsule (210 mg) contained no less than 1.0 × 10^7^CFU viable bacteria. The Rif group received rifaximin at 200 mg twice a day. The Lac group received lactulose treatment to maintain defecation 2–3 times per day. Blood and stool samples were collected at entry and 4 weeks, and PHES tests were also performed.

### Laboratory tests

Blood samples for assessment of blood ammonia, liver function, kidney function, and anticoagulant function were sent to the Laboratory of the First Affiliated Hospital of Anhui Medical University for measurements within half an hour after collection. Child–Turcotte–Pugh (CTP) and model for end-stage liver disease (MELD) scores were calculated using standard clinical and laboratory measures.

### 16S rDNA gene sequencing

All stool samples were collected with sterile cotton swabs and placed in a 2 ml sterile sampling tubes. Samples were then sent to Novogene sequencing center in Tianjin, China for DNA extraction and sequencing. The genomic DNA was extracted with QIAamp Fast DNA Stool Mini Kit (Qiagen, Hilden, Germany), and 16S rDNA PCR primers (341F, CCTAYGGGRBGCASCAG; 806R, GGACTACNNGGGTATCTAAT) were used to amplify 16S rDNA’s V3–V4 hypervariable region using total DNA from each sample as a PCR template. The library was constructed with TruSeq DNA PCR-Free Sample Preparation Kit, and sequencing used the NovaSeq system (Illumina) based on the 2 × 250-base pair protocol.

### Bioinformatic analysis of 16S rDNA gene sequencing

First, the primer region for 16S rDNA gene sequencing raw data was removed with Cutadapt (version 1.18). The paired sequences were merged with Vsearch (version 2.14.1) with default parameters (Rognes et al., 2016). Next, the merged reads were analyzed with QIIME2 (version 2019.10) (Bolyen et al., 2019). Deblur was utilized to filter low-quality merged reads and to construct a feature table (100% identity) (Amir et al., 2017). The samples with a total abundance (total number of sequences obtained from the sample) >16,000, features with a total abundance >10 and observed in at least five samples were reserved for subsequent analysis. The Greengenes 16S database (version 13.8) was used for taxonomy assignment of sequence datasets and performed by the QIIME2 plugin feature sklearn classifier (McDonald et al., 2012; Bokulich et al., 2018). Alpha and beta diversities were assessed with rarefied feature table using QIIME2; the metaCyc metabolic pathways of bacteria were predicted with PICRUSt2 (version 2.3.0) (Douglas et al., 2020). The random forest model was conducted based on genera level with relative abundance >0.1% using R package RandomForest. All figures were visualized using R software (v4.1.0).

### Statistical analysis

Normally distributed data were compared by the paired t test, ANOVA, or chi-square tests and expressed as mean (SD). Non-normally distributed data were compared by the Kruskal–Wallis rank sum tests. Chi-square test was used to compare the efficacy of different drugs. The differences in Alpha diversity were assessed using an ANOVA test for multiple groups. Comparisons of dissimilarities between multiple groups were performed using the permutational multivariate analysis of variance test (PERMANOVA). The differential abundance levels of taxa were compared using LEfSe and DESeq2 (Segata et al., 2011; Love et al., 2014). And differential metabolic features between two groups were compared with t test. All *p* values <0.05 after multiple comparisons correction using false discovery rate method were considered significantly different.

## Results

### PHES reference ranges

A total of 200 healthy volunteers were assessed, including 109 males and 91 females, aged 47.7 ± 10.5 years, with 10.7 ± 4.2 years of education. After grouping according to age and education level, the experimental reference ranges of the five sub-items in each group were shown in Supplementary Table 1.

### Clinical outcomes

**FIGURE 1:**
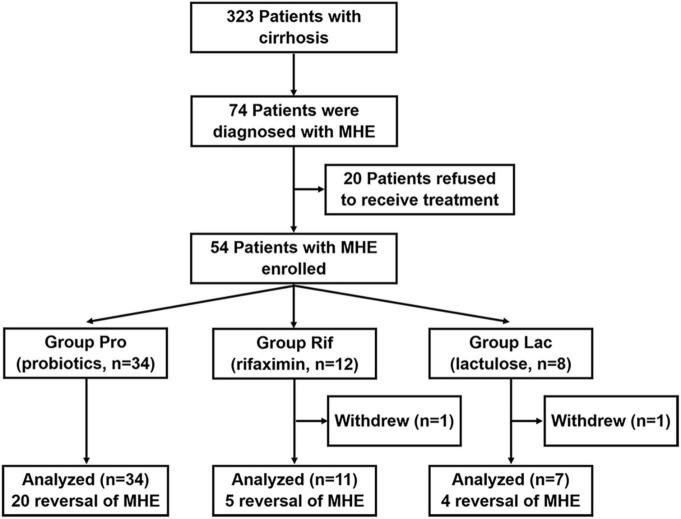
Flow of patients of treatment for MHE.

**FIGURE 2:**
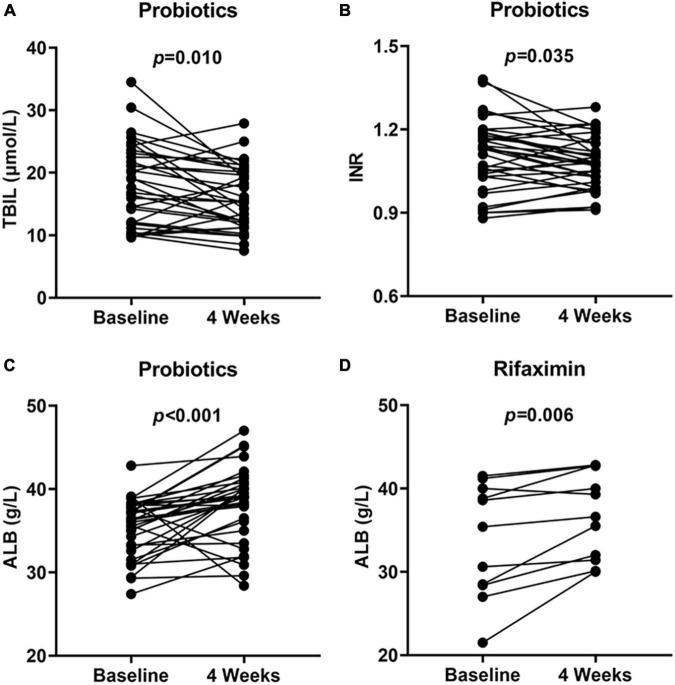
Hematological indexes at baseline and 4 weeks showed significant improvement. TBIL **(A)**, INR **(B)**, and ALB **(C)** performance at baseline and 4 weeks in probiotics group and ALB **(D)** performance at baseline and 4 weeks in rifaximin group. TBIL, total bilirubin; INR, international normalized ratio; ALB, serum albumin.

A total of 323 patients with cirrhosis were screened for PHES. Of these patients, 74 were diagnosed with MHE, indicating an MHE incidence of 22.9%. Totally 52 patients successfully completed the treatment and provided samples of blood and stool (Figure 1). Their baseline clinical characteristics are presented in Table 1. The recovery rates of Pro, Rif, and Lac group were 58.8% (20/34), 45.5% (5/11), and 57.1% (4/7), respectively. There was no statistically significant difference among probiotics, rifaximin, and lactulose in MHE recovery (*p* = 0.843). The 20 MHE patients that refused to receive the treatment were not tested by PHES again. Unfortunately, the HE exacerbations were found in 2 of them within 6 months follow-up. There was no parallel among the MHE patients received the treatment. Hematological indexes were compared before and after treatment in different groups, and the results were shown in Table 2. Increased serum albumin, reduced serum total bilirubin (TBIL), and international normalized ratio (INR) were detected after probiotic treatment, and increased serum albumin levels were found after rifaximin treatment (Figure 2). No statistically significant differences were found in other indexes. Overall patients were diagnosed as response or non-response according to whether MHE was recovered or not. We found that non-responsive patients had higher baseline INR (1.16 ± 0.11 vs. 1.08 ± 0.13, *p* = 0.026) and MELD (5.11 ± 3.11 vs. 2.77 ± 2.73, *p* = 0.007) as compared with responsive patients. Particularly, patients did not responded to probiotics treatment showed higher baseline TBIL, INR, and MELD performance as compared with patients who responded (Table 3).

**TABLE 1:** Baseline data of MHE patients.

Clinical characteristics	Pro (*n* = 34)	Rif (*n* = 11)	Lac (*n* = 7)	*p*
Age (year)	55.68 ± 6.00	56.00 ± 6.91	56.71 ± 7.48	0.924
Sex (male/female)	19/15	7/4	4/3	0.917
Education (year)	8.44 ± 3.56	8.91 ± 3.73	8.71 ± 4.96	0.934
Ammonia (μmol/L)	22.82 ± 4.57	24.82 ± 8.55	17.40 ± 5.46	0.065
TBIL (μmol/L)	18.25 ± 6.54	20.45 ± 8.03	17.14 ± 5.24	0.57
PT (s)	14.27 ± 1.62	15.39 ± 1.07	13.64 ± 1.50	0.055
PHES	−5.06 ± 1.15	−5.09 ± 1.04	−4.86 ± 1.21	0.843
CTP	5.88 ± 0.84	7.00 ± 1.67	5.80 ± 0.84	0.086
MELD	3.42 ± 2.87	5.16 ± 3.02	3.85 ± 4.61	0.278

Cases lost to follow-up have been excluded. Data is presented as mean ± SD unless mentioned otherwise. Comparisons performed using ANOVA, chi-square tests or Kruskal–Wallis rank sum tests. TBIL, total bilirubin; PT, prothrombin time; PHES, psychometric hepatic encephalopathy score; CTP, Child–Turcotte–Pugh; MELD: model for end-stage liver disease.

**TABLE 2:** Changes of blood ammonia and liver function before and after treatment.

	Pro			Rif			Lac		
	Baseline	4 weeks	*p*	Baseline	4 weeks	*p*	Baseline	4 weeks	*p*
Ammonia (μmol/L)	22.82 ± 4.57	21.21 ± 4.22	0.136	24.82 ± 8.55	20.73 ± 7.39	0.065	17.40 ± 5.46	18.80 ± 4.66	0.575
AST (U/L)	33.26 ± 9.81	33.03 ± 10.53	0.903	34.55 ± 6.19	37.27 ± 9.57	0.446	36.80 ± 15.50	34.20 ± 14.24	0.545
ALT (U/L)	24.76 ± 9.90	23.44 ± 8.70	0.464	23.64 ± 7.88	25.27 ± 11.54	0.639	21.60 ± 7.80	23.40 ± 4.83	0.405
TBIL (μmol/L)	18.25 ± 6.54	15.90 ± 5.01	0.010	20.45 ± 8.03	18.73 ± 6.50	0.251	17.14 ± 5.24	18.87 ± 9.67	0.712
ALB (g/L)	35.42 ± 3.43	38.25 ± 4.43	<0.001	33.77 ± 6.84	36.65 ± 5.18	0.006	35.38 ± 3.49	38.14 ± 4.58	0.138
PT (s)	14.27 ± 1.62	14.14 ± 1.56	0.573	15.39 ± 1.07	15.19 ± 1.48	0.64	13.64 ± 1.50	14.08 ± 1.51	0.464
INR	1.11 ± 0.13	1.08 ± 0.09	0.035	1.17 ± 0.13	1.28 ± 0.27	0.271	1.07 ± 0.10	1.10 ± 0.10	0.53
CRE (μmol/L)	58.88 ± 12.72	59.55 ± 12.95	0.702	64.14 ± 14.69	62.71 ± 7.24	0.736	65.22 ± 17.74	67.98 ± 21.25	0.39
CTP	5.88 ± 0.84	5.59 ± 0.78	0.106	7.00 ± 1.67	6.64 ± 1.43	0.564	5.80 ± 0.84	5.60 ± 0.89	0.65
MELD	3.42 ± 2.87	2.78 ± 2.37	0.141	5.16 ± 3.02	5.75 ± 3.50	0.659	3.85 ± 4.61	4.61 ± 5.61	0.634

Data is presented as mean ± SD unless mentioned otherwise. Comparisons performed using paired *t*-tests or Kruskal–Wallis rank sum tests as appropriate. AST, aspartate aminotransferase; ALT, alanine aminotransferase; ALB, serum albumin; TBIL, total bilirubin; PT, prothrombin time; INR, international normalized ratio; CRE, creatinine; CTP, Child–Turcotte–Pugh; MELD, model for end-stage liver disease.

**TABLE 3:** Predictors of non-response to therapy in the three groups.

	Overall			Pro			Rif			Lac		
	Response	Non-response	*p*	Response	Non-response	*p*	Response	Non-response	*p*	Response	Non-response	*p*
Age	55.66 ± 6.15	56.17 ± 6.56	0.77	55.60 ± 6.29	55.79 ± 5.79	0.931	56.20 ± 6.91	55.83 ± 7.57	0.936	55.25 ± 6.18	58.67 ± 10.02	0.598
Ammonia (μmol/L)	23.74 ± 4.54	21.52 ± 7.18	0.191	24.10 ± 4.48	21.00 ± 4.19	0.05	23.60 ± 5.59	25.83 ± 10.89	0.689	20.50 ± 2.12	15.33 ± 6.43	0.371
AST (U/L)	33.07 ± 9.86	34.87 ± 9.56	0.518	31.65 ± 9.61	35.57 ± 9.97	0.257	36.20 ± 5.50	33.17 ± 6.88	0.447	39.50 ± 21.92	35.00 ± 15.10	0.798
ALT (U/L)	25.26 ± 10.25	22.96 ± 7.82	0.383	24.55 ± 11.16	25.07 ± 8.15	0.883	29.20 ± 6.10	19.00 ± 6.13	0.022	22.50 ± 10.61	21.00 ± 8.00	0.866
TBIL (μmol/L)	16.96 ± 6.32	20.57 ± 6.81	0.058	16.03 ± 5.67	21.42 ± 6.58	0.016	21.89 ± 8.16	19.24 ± 8.47	0.613	13.99 ± 1.90	19.23 ± 6.06	0.339
ALB (g/L)	34.21 ± 4.61	36.04 ± 3.90	0.141	34.85 ± 3.39	36.24 ± 3.44	0.248	31.44 ± 8.41	35.72 ± 5.20	0.327	34.85 ± 1.77	35.73 ± 4.73	0.824
PT (s)	14.06 ± 1.66	14.92 ± 1.36	0.055	13.87 ± 1.67	14.86 ± 1.41	0.079	15.26 ± 1.41	15.50 ± 0.81	0.731	13.05 ± 0.78	14.03 ± 1.91	0.554
INR	1.08 ± 0.14	1.16 ± 0.11	0.026	1.07 ± 0.13	1.16 ± 0.11	0.039	1.15 ± 0.15	1.18 ± 0.13	0.780	1.00 ± 0.06	1.12 ± 0.09	0.197
CRE (μmol/L)	58.39 ± 12.95	63.34 ± 14.20	0.204	58.51 ± 12.39	59.41 ± 13.63	0.841	58.38 ± 15.02	68.93 ± 13.80	0.256	57.30 ± 23.19	70.50 ± 15.99	0.496
PHES	−4.97 ± 1.02	−5.13 ± 1.25	0.747	−5.00 ± 1.08	−5.14 ± 1.29	0.839	−5.20 ± 0.84	−5.00 ± 1.26	0.77	−4.50 ± 1.00	−5.33 ± 1.53	0.329
CTP	6.11 ± 1.09	6.13 ± 1.25	0.959	5.95 ± 0.76	5.79 ± 0.97	0.383	7.00 ± 1.87	7.00 ± 1.67	0.779	5.50 ± 0.71	6.00 ± 1.00	0.543
MELD	2.77 ± 2.73	5.11 ± 3.11	0.007	2.52 ± 2.40	4.70 ± 3.09	0.027	4.44 ± 3.06	5.76 ± 3.13	0.500	1.09 ± 5.13	5.68 ± 4.09	0.342

Data is presented as mean ± SD unless mentioned otherwise. Comparisons performed using *t*-tests or Kruskal–Wallis rank sum tests as appropriate. AST, aspartate aminotransferase; ALT, alanine aminotransferase; ALB, serum albumin; TBIL: total bilirubin; PT, prothrombin time; INR, international normalized ratio; CRE, creatinine; CTP, Child–Turcotte–Pugh; MELD, model for end-stage liver disease.

Although there were no serious adverse events, 3 patients had minor adverse reactions. The first patient developed abdominal pain after taking lactulose for 1 week, and the second patient had constipation after taking rifaximin for 1 week. The forementioned signs of both patients subsided after discontinuation, and these cases were withdrawn from the study. Another patient developed diarrhea after taking lactulose, which resolved after dose adjustment as instructed.

### Characteristics of the gut microbiota in MHE patients

**FIGURE 3:**
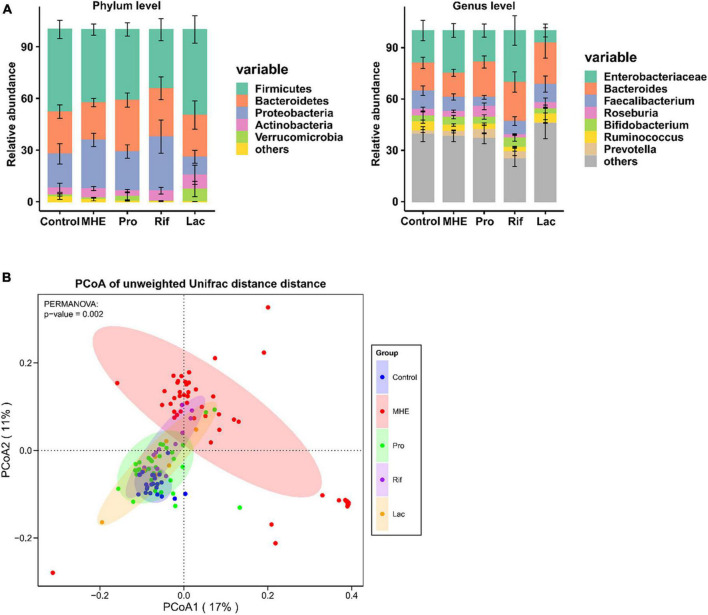
The gut microbiota in MHE patients and controls. **(A)** Compositions of intestinal microbiota in different groups. **(B)** PCoA based on unweighted UniFrac matrix of bacterial taxonomy in all patients and control individuals.

**FIGURE 4:**
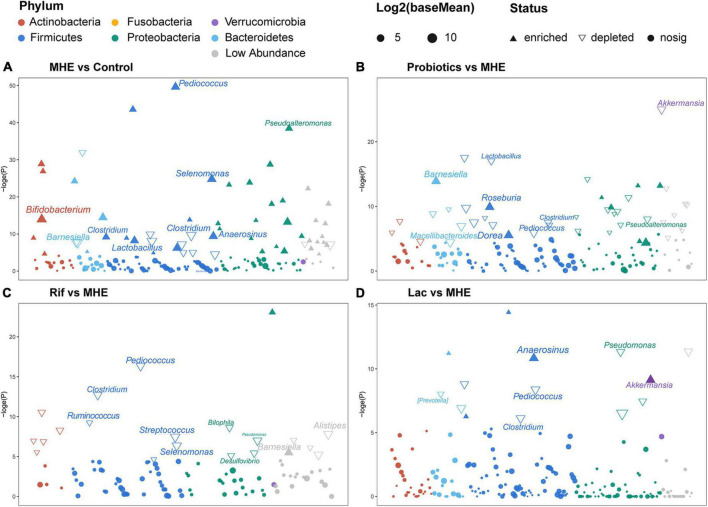
Differential taxonomic features based on DESeq2 analysis. Changes in relative abundance of the gut microbiota between MHE patients and controls **(A)**, MHE patients before and after probiotics treatment **(B)**, MHE patients before and after rifaximin treatment **(C)**, and MHE patients before and after lactulose treatment **(D)**. Log2(baseMean): the log base 2 of base mean, the base mean is the mean of normalized counts of all samples, normalizing for sequencing depth. –loge(P): the negative of log base e of *p* value.

The most abundant bacterial phyla and the top 7 genera in stool samples from healthy controls and MHE patients before and after treatment were shown in Figure 3A. The relative abundance at the phylum level showed the enrichment of Proteobacteria and the lack of Bacteroidetes, and the relative abundance at the genus level showed the enrichment of enterobacteriaceae and the lack of Bacteroidetes in in MHE patients as compared with control individuals before treatment. Comparing the α-diversity of the gut microbiota between control individuals and MHE patients, no significant differences were found in Chao1 index, observed OTUs, Shannon index, and Faith_PD (Supplementary Figure 1A). Principal coordinate analysis based on unweighted UniFrac matrix of bacterial taxonomy showed that taxonomic compositions in MHE patients were distinct from those of healthy individuals before treatment (Figure 3B). However, no significant difference were found in principal coordinate analysis based on weighted UniFrac and Bray-Curtis matrix (Supplementary Figure 1B). DESeq2 analysis was performed to compare differential taxa between MHE and control cases. The results showed the enrichment of *Pediococcus*, *Selenomonas*, *Anaerosinus*, *Lactobacillus*, *Pseudomonas*, and *Bifidobacterium*, and the lack of *Barnesiella* in MHE patients (Figure 4A). LEfSe analysis showed similar results (Supplementary Figure 2A).

### Alterations of taxonomic and metabolic compositions following treatment

The top 5 relative abundance at the phylum level showed the trend of increased Bacteroidetes in MHE after treatment, regardless of the treatment methods. The similar result was found at the genus level (Figure 3A). We analyzed the bacterial diversity in MHE patients before after treatment. Interestingly, α-diversity did not change significantly after treatment, but principal coordinate analysis based on unweighted UniFrac matrix suggested that the taxonomic compositions in MHE patients were significantly changed after treatment, and gradually tended to those of healthy individuals (Figure 3B and Supplementary Figure 1A). Accordingly, differential taxa were determined in MHE patients before and after treatment. The relative abundance of *Pediococcus*, *Lactobacillus*, *Akkermansia*, and *Macellibacteroides* decreased, while the abundance of *Barnesiella*, *Dorea*, and *Roseburia* increased after probiotics treatment (Figure 4B). Rifaximin treatment reduced the abundance of *Clostridium*, *Pediococcus*, *Ruminococcus*, *Streptococcus*, *Selenomonas*, *Biophila*, *Desulfovibrio*, and *Pseudomonas*, and increased the abundance of *Barnesiella* (Figure 4C). The Lac group showed lower abundance of *Pediococcus*, *Clostridium*, and *Pseudomonas*, and higher abundance of *Anaerosinus* and *Akkermansia* (Figure 4D). LEfSe analysis showed similar results (Supplementary Figures 2B–D).

**FIGURE 5:**
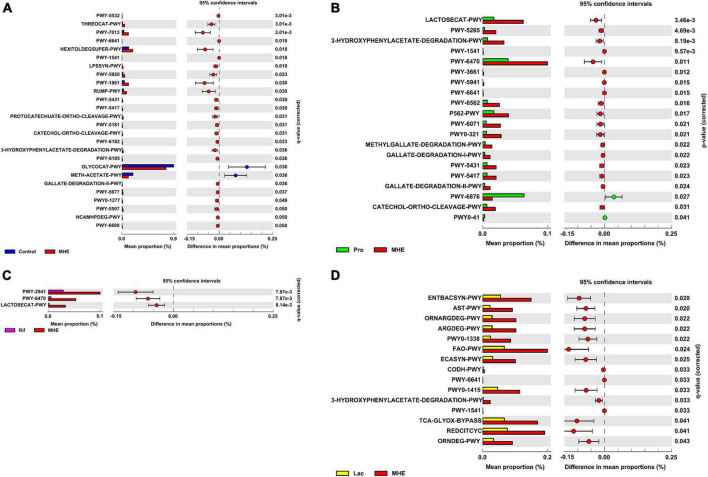
Differential metabolic features in patients before and after treatment. Comparison of differential metabolic features between MHE patients and healthy controls **(A)**, MHE patients before and after probiotics treatment **(B)**, MHE patients before and after rifaximin treatment **(C)**, and MHE patients before and after lactulose treatment **(D)**. *q*-value, adjusted *p*-value by Benjamini–Hochberg method. If *q*-value is not significant, *p*-value is used.

PICRUSt2 was used to predict the metabolic pathways possessed by the gut microbiota based on 16S rDNA gene sequencing. The metabolic pathways were compared, and no obvious changes were found between the MHE and the control group (Figure 5A). However, the metabolic pathways in MHE patients before and after probiotics treatment showed difference. Downregulated pathways after probiotics treatment included glycometabolism (LACTOSECAT-PWY, PWY-6470, and PWY-5265) and degradation of aromatic compounds (3-HYDROXYPHENYLACETATE-DEGRADATION-PWY, PWY-6071, PWY0-321, METHYLGALLATE-DEGRADATION-PWY, GALLATE-DEGRADATION-I-PWY, PWY-5431, PWY-5417, GALLATE-DEGRADATION-II-PWY, and CATECHOL-ORTHO-CLEAVAGE-PWY), and PWY-6876 was upregulated. There was no significant pathway change before and after rifaximin treatment. After lactulose treatment, degradation pathways of arginine and ornithine (ORNDEG-PWY, ARGDEG-PWY, ORNARGDEG-PWY, and AST-PWY) showed a downward trend (Figures 5B–D). The description of all predicted metabolic pathways were listed in Supplementary Table 2.

### The key taxa and their correlations with biomarkers

**FIGURE 6:**
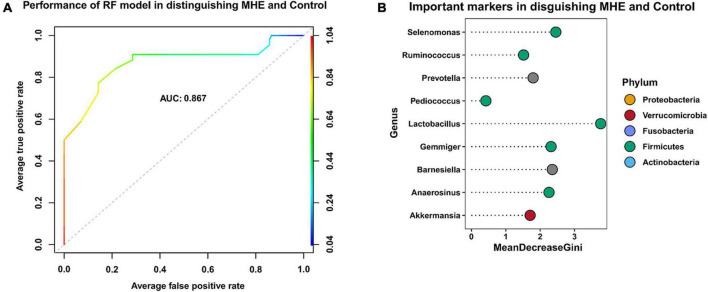
Key taxa related to MHE. Receiver operating curve (ROC)-analysis showed the performance of random forest model in distinguishing MHE and control group **(A)**. The top 9 important taxa selected by random forest model **(B)**.

To identify key taxa involved in MHE, we compared the relative abundance at the genus level between MHE patients and control individuals. The results showed the enrichment of *Selenomonas*, *Anaerosinus*, *Lactobacillus*, *Pediococcus*, and *Prevotella*, and the lack of *Ruminococcus*, *Barnesiella*, and *Gemmiger*. Using a combination of different taxa to predict MHE, an area under the curve of 0.867 was obtained (Figure 6).

**FIGURE 7:**
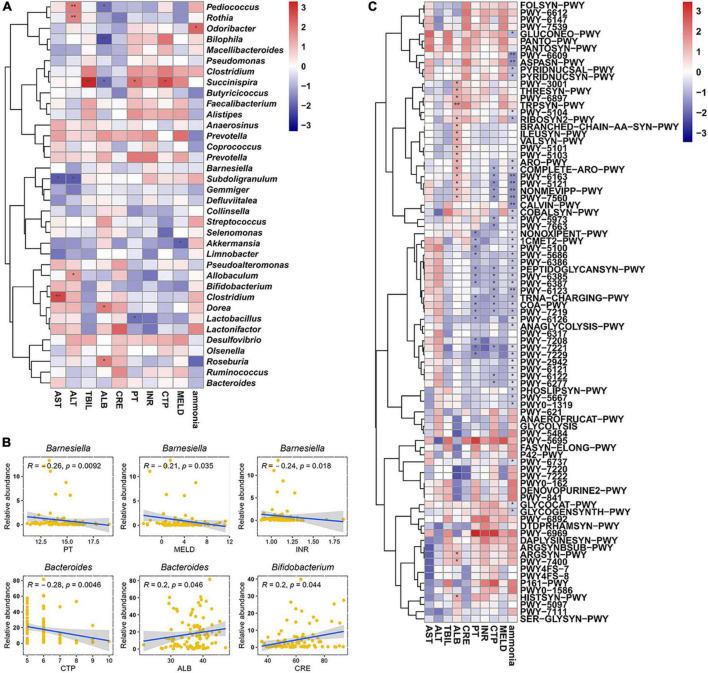
Analysis of correlation. **(A)** Correlation coefficients between biomarkers and gut microbiota at genus level. **(B)** Key genus significantly correlated with different biomarkers. **(C)** Correlation coefficients between biomarkers and metabolic features. The Spearman correlation coefficients were calculated and labeled in the figure. AST, aspartate aminotransferase; ALT, alanine aminotransferase; ALB, serum albumin; TBIL, total bilirubin; PT, prothrombin time; INR, international normalized ratio; CRE, creatinine; CTP, Child–Turcotte–Pugh; MELD, model for end-stage liver disease. The Spearman correlation coefficients were calculated and labeled in the figure. Statistically significances are indicated: *p < 0.05, **p < 0.01.

Subsequently, Spearman correlation coefficients between specific genus and biomarkers of cirrhosis were calculated. *Pediococcus*, *Rothia*, and *Allobaculum* were positively correlated with alanine aminotransferase; *Bilophila* was negatively correlated with serum albumin; *Odoribacter* was positively correlated with ammonia; *Succinispira* was positively correlated with TBIL, prothrombin time (PT), and CTP, and negatively correlated with serum albumin; *Subdoligranulum* was negatively correlated with alanine aminotransferase and aspartate aminotransferase; *Akkermansia* was negatively correlated with MELD; *Clostridium* was positively correlated with aspartate aminotransferase; *Dorea* and *Roseburia* were positively correlated with serum albumin; *Lactobacillus* was negatively correlated with PT (Figure 7A). Particularly, *Barnesiella* was negatively correlated with PT (*p* = 0.0092), MELD (*p* = 0.035), and INR (*p* = 0.018) performance after correction; *Bacteroides* was negatively correlated with CTP score (*p* = 0.0046) and positively correlated with serum albumin level (*p* = 0.046) after correction; *Bifidobacterium* was positively correlated with serum creatinine level (*p* = 0.044) (Figure 7B). Spearman correlation coefficients between specific genus and metabolic pathways had shown in Figure 7C.

## Discussion

This study revealed that probiotics, rifaximin, and lactulose were safe and effective in the treatment of MHE, with reverse rates of 58.8% (20/34), 45.5% (5/11), and 57.1% (4/7), respectively. The Chi-square test showed that there was no significant difference in efficacy among the three drugs. Previous studies found that lactulose and probiotics are effective for secondary prophylaxis of HE in patients with cirrhosis, and probiotics may be non-inferior to lactulose (Agrawal et al., 2012; Pratap Mouli et al., 2015). Another study showed rifaximin and lactulose have comparable efficacy in reversing MHE (Sidhu et al., 2016). These studies revealed that probiotics, rifaximin, and lactulose were safe and effective in the treatment of MHE.

In addition, we found that probiotics and rifaximin improved liver function to some extent, including serum TBIL, serum albumin, and INR, which were corroborated in previous findings. Dhiman et al. (2014) found that CTP and MELD were improved significantly from baseline to 6 months in the probiotic group. In addition, there was a significant improvement in serum bilirubin after rifaximin therapy (Bajaj et al., 2013). However, we found no significant effect of lactulose on liver function. The possible reason is that its main role is to regulate intestinal pH to maintain an acidic environment, and to play a cathartic role by stimulating colon peristalsis. Meanwhile, it inhibits glutamylase activity and interferes with ammonia metabolism to realize the reversal of MHE (Dhiman, 2012). Interestingly, there was no significant difference in blood ammonia after treatment. Although some studies had found that probiotics could reduce blood ammonia (Lunia et al., 2014; Pratap Mouli et al., 2015), other reports showed no change in blood ammonia levels, or even increased amounts after treatment (Mittal et al., 2011; Bajaj et al., 2014a). These findings indicated that ammonia levels were not a direct guide to the treatment of patients with HE (Haj and Rockey, 2020).

Patients with cirrhosis commonly have an altered gut microbiome with reduced proportions of beneficial, autochthonous taxa, including Lachnospiraceae, Ruminococcaceae, and Clostridiales XIV. There is a relative overgrowth of potentially pathogenic bacteria such as Enterobacteriaceae, Staphylococcaceae, and Enterococcaceae, whose abundance levels correlate with disease progression and endotoxemia (Chen et al., 2011; Bajaj et al., 2012; Bhat et al., 2016). Meanwhile, dysbiosis is gradually aggravated with the deterioration of liver function, especially in patients with HE (Bajaj et al., 2014b). This was confirmed in this study, the PCoA results suggested the presence of gut dysbiosis despite no significant change in α diversity. This study also demonstrated that the relative abundance levels of *Bacteroides* and *Barnesiella* in intestinal microbiota were lower in MHE, while *Lactobacillus* had higher levels. These findings corroborated previous studies of the gut microbiota, in which the relative abundance levels of Lactobacillaceae in stool and saliva samples were higher in MHE regardless of diagnostic methods (Bajaj et al., 2019). The incidence rate of MHE was significantly lower in the *Bacteroides*-dominant group than in the non-dominant group (Haraguchi et al., 2019), and was respectively associated with HE recurrence and overall survival during the subsequent one-year follow-up (Sung et al., 2019). In this study, we also found the relative abundance of *Bacteroides* in MHE patients showed the rising trend after treatment. This indicated that *Bacteroides* may be related to the development and outcome of MHE. Another study reported that *Barnesiella* was positively associated with good cognition and involved in carbohydrate fermentation, competitive inhibition of pathogenic bacteria, and immunoregulation (Meyer et al., 2022). These parameters may be potential promoting factors in MHE.

The gut microbiota showed different changes after treatment. There was no significant difference in α-diversity of gut microbiota after treatment with probiotics, rifaximin, or lactulose, suggested that α-diversity might not be a key factor in the pathogenesis and treatment of MHE. However, PCoA based on unweighted UniFrac matrix showed that all therapies improved dysbiosis, rendering the microbiota closer to that of healthy individuals. Probiotics can improve the abundance of *Barnesiella*, *Roseburia*, and *Bacteroides*, and can reduce the abundance of *Lactobacillus* in MHE patients. As mentioned above, *Barnesiella* was positively associated with good cognition. Song et al. (2022) found *Roseburia hominis* alleviates neuroinflammation by producing propionate and butyrate. It has also been reported that the family Bacteroidaceae is negatively correlated with systemic and neural inflammation in cirrhotic mice, and *Bacteroides* species play a vital role in bile duct ligation-evoked cholestatic liver disease-related cognitive dysfunction (Kang et al., 2016; Yang et al., 2022). This may partly explain the improvement of cognitive function by probiotics. The Rif group showed an overall downward trend. Changes in the gut microbiota were analyzed in three previous studies assessing rifaximin treatment in MHE. The first two studies found that rifaximin decreased bacterial richness, but the effects on particular species were not observed; in addition, circulating markers of inflammation were unaltered by rifaximin treatment (Kimer et al., 2018; Schulz et al., 2019). This may be due to the broad-spectrum bactericidal effect of rifaximin. However, the third study showed no significant microbial changes besides modestly decreased Veillonellaceae and increased Eubacteriaceae. Rifaximin treatment resulted in a significant reduction in network connectivity and clustering on correlation networks (Bajaj et al., 2013). Rifaximin treatment was significantly associated with prolonged overall survival and reduced risk of spontaneous bacterial peritonitis, variceal bleeding and recurrent HE (Kang et al., 2017). This may be due to different intervention cycles, doses, and/or statistical and analytical methods. *Akkermansia* and *Anaerosinus* amounts were higher in the Lac group, while *Pediococcus* and *Pseudomonas* levels were lower. *Akkermansia* has the functions of intestinal barrier protection, anti-inflammation, and immune regulation (Zhang et al., 2019). Significant differences were found between lactulose responders and non-responders in Actinobacteria, Bacteroidetes, Firmicutes, and Proteobacteria (Wang et al., 2019), which may be related to the efficacy of lactulose.

We used PICRUSt2 to predict the metabolic pathways possessed by the gut microbiota, and glycometabolism and degradation of aromatic compounds were decreased after probiotics treatment. Aromatic amino acids and branched-chain amino acids compete for entry across the blood-brain barrier via the same transporter leading to the neuro-depression observed in HE (Gluud et al., 2017). Another potential mechanism by which probiotics recover MHE may be achieved by altering the accumulation of aromatic amino acids. After lactulose treatment, several metabolic pathways associated with the degradation of arginine and ornithine were downregulated. It means that levels of ornithine may be higher in the lactulose group, while ornithine enhances ammonia metabolism and was used in the treatment of HE (Goh et al., 2018).

To identify key taxa involved in MHE, we compared the relative abundance at the genus level between MHE patients and control individuals. The results showed *Selenomonas*, *Anaerosinus*, *Lactobacillus*, *Pediococcus*, *Prevotella*, *Ruminococcus*, *Barnesiella*, and *Gemmiger* were the key taxa to distinguish MHE or not. We used a combination of different taxa to predict MHE (area under the curve of 0.867), which is expected to be used in the auxiliary diagnosis of MHE.

The comparison of responsive and non-responsive patients showed lower MELD and INR in responsive patients, especially in Pro group. Coincidentally, we found that *Barnesiella* was negatively correlated with MELD and INR. This may provide a new idea for studying the role of *Barnesiella* in MHE.

This study had several limitations. First of all, the sample size was not large, especially for the Rif and Lac group. This may has impact on the confidence of the conclusion. We used statistical methods to minimize the impact of small sample size. If possible, we plan to expand the number of enrolled patients in subsequent study. In addition, the patients were followed up for a short time, with no long-term follow-up and no placebo group was included. Furthermore, only stool specimens were collected, and mucous membranes or other specimens were not obtained. Finally, metabolomics analysis was not carried out, and the underlying mechanism could not be further explored.

In conclusion, probiotics, rifaximin, and lactulose were safe and effective in the treatment of MHE. Liver function and gut dysbiosis were improved to some extent in the current patients. There were significant changes in both taxonomic and metabolic parameters after treatment. These findings might not only provide a valuable reference for pathogenesis studies but also help improve therapeutic strategies for MHE.

## Data availability statement

The datasets presented in this study can be found in online repositories. The names of the repository/repositories and accession number(s) can be found below: https://www.ncbi.nlm.nih.gov/, PRJNA898090.

## Ethics statement

The studies involving human participants were reviewed and approved by the Ethics Committee of the First Affiliated Hospital of Anhui Medical University (No. PJP2020-15-24). The patients/participants provided their written informed consent to participate in this study.

## Author contributions

XC and JG conceived and designed the trial. M-WW and W-JM collected the data. M-WW and JG performed the bioinformatics and statistical analysis. X-HM and YW drafted the manuscript. Y-FX and W-JM edited the manuscript. All authors read and approved the final manuscript.
