# Supplementary material for: Comparison of the effects of probiotics, rifaximin, and lactulose in the treatment of minimal hepatic encephalopathy and gut microbiota
Source: Front Microbiol. 2023 Mar 24;14:1091167. doi: 10.3389/fmicb.2023.1091167 (PMC10080009; doi:10.3389/fmicb.2023.1091167)
Supplement: Supplementary file 1 [file Data_Sheet_1.docx]

Supplementary Material

# Supplementary Data

Supplementary Material remains metadata (Wang_metadata. xlsx)

# Supplementary Figures and Tables

**
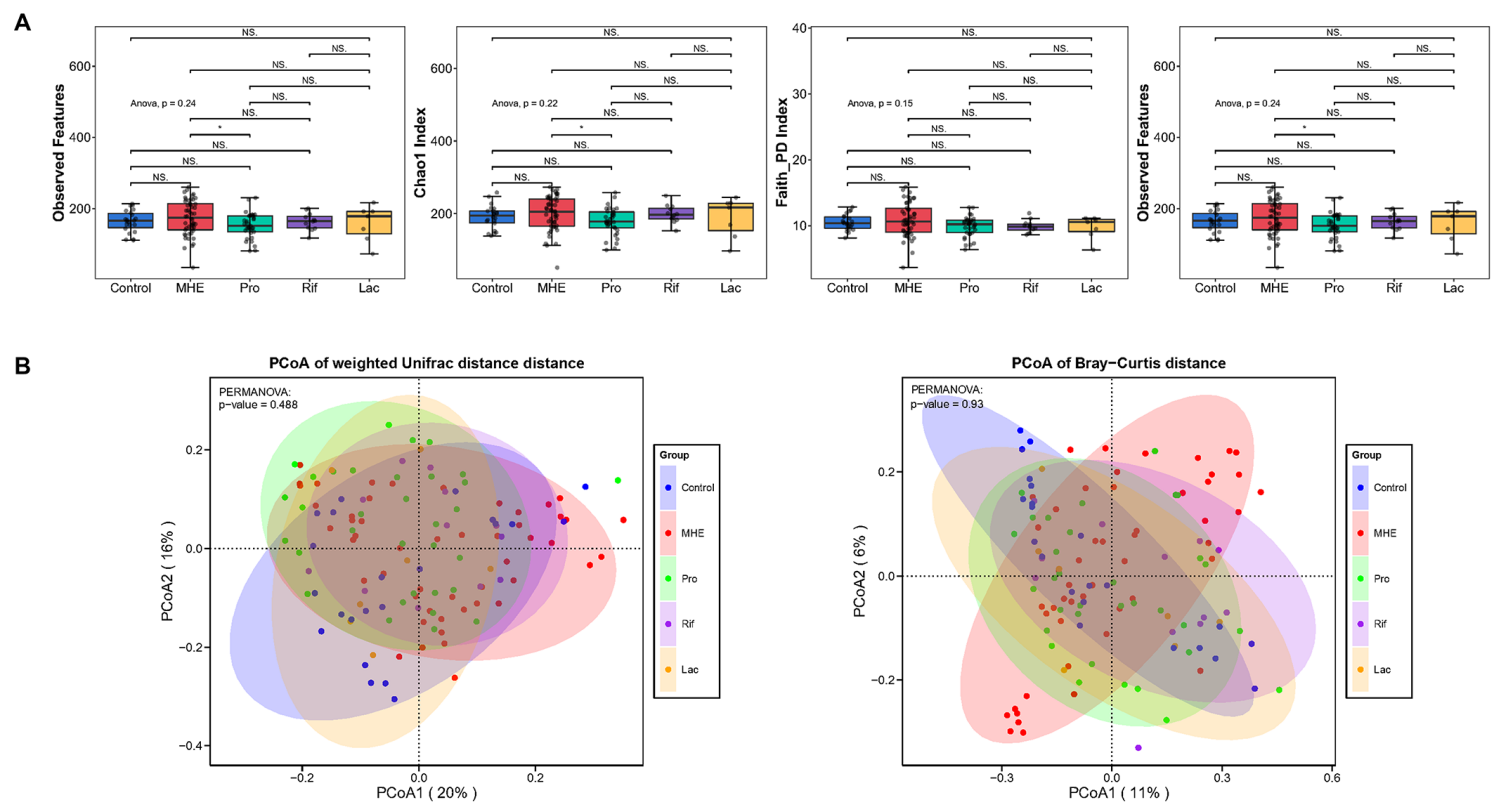
**

**Supplementary Figure 1** Diversities of gut microbiota in MHE patients and controls. (A) Alpha diversities of all patients and control individuals. (B) PCoA based on weighted UniFrac matrix and Bray-Curtis matrix of bacterial taxonomy in all patients and control individuals.

**
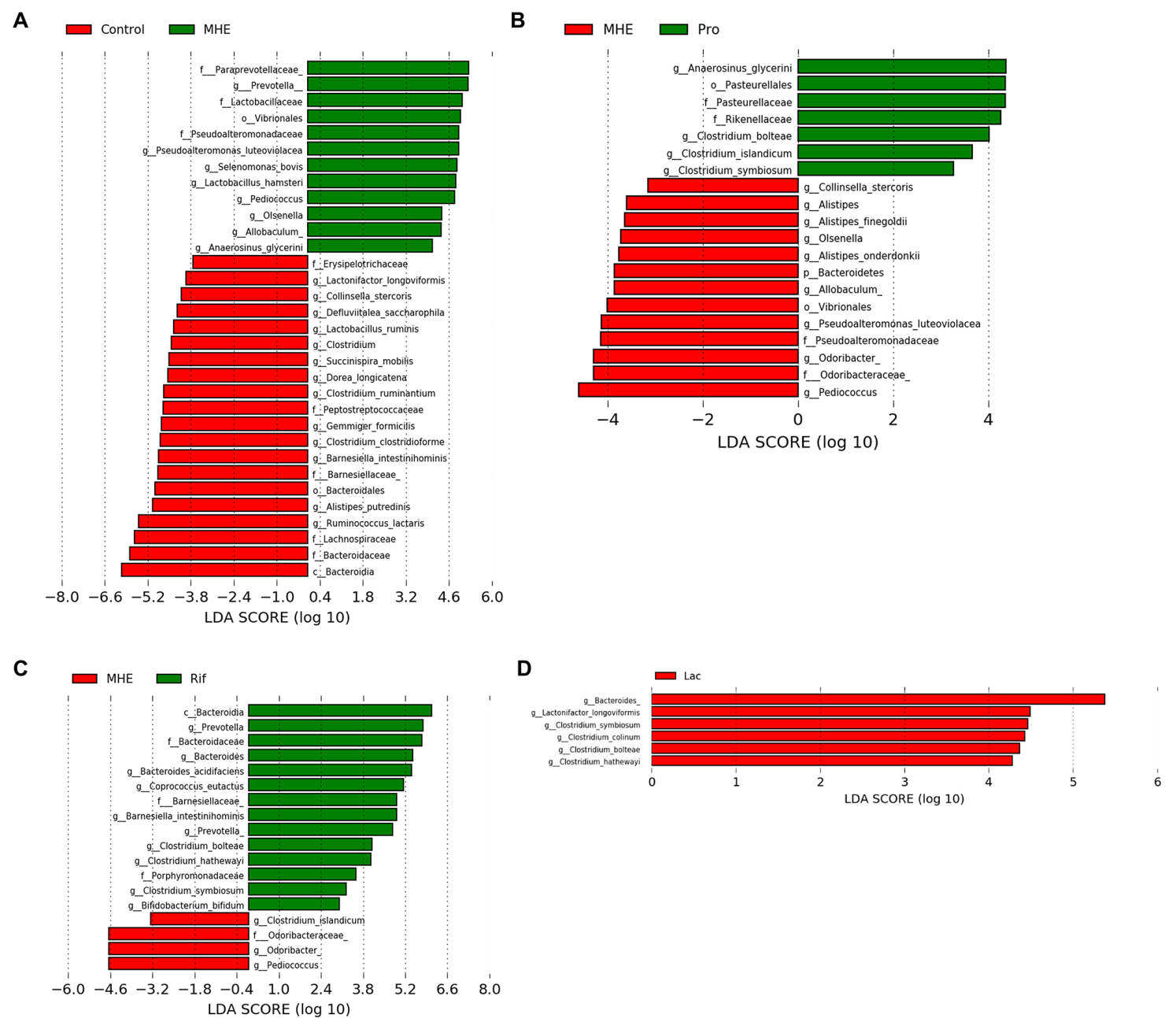
**

**Supplementary Figure 2** Differential taxonomic features based on LEfSe analysis. Histograms of the LDA scores for taxonomic features differentially abundant between MHE patients and controls (A), MHE patients before and after probiotics treatment (B), MHE patients before and after rifaximin treatment (C), and MHE patients before and after lactulose treatment (D).

**Supplementary Table 1. PHES test reference values for different populations**

| Age/education years | NCT-A (s) | NCT-B/C (s) | DST | LTT (s) | SDT (s) |
| --- | --- | --- | --- | --- | --- |
|  |  |  |  |  |  |
| 31-40/0-9 | 31.58±5.03 | 44.13±8.14 | 54.50±8.82 | 47.33±7.87 | 47.88±10.37 |
| 31-40/＞9 | 28.73±6.90 | 41.15±8.85 | 63.82±9.47 | 44.55±9.98 | 39.82±10.04 |
| 41-50/0-9 | 42.90±8.87 | 58.17±9.12 | 41.17±8.39 | 50.87±10.72 | 43.67±5.76 |
| 41-50/＞9 | 33.31±10.21 | 45.44±13.24 | 57.41±10.11 | 47.31±10.68 | 45.53±14.52 |
| 51-60/0-9 | 55.96±11.75 | 72.00±10.13 | 31.70±7.44 | 62.74±8.04 | 58.41±10.09 |
| 51-60/＞9 | 47.67±11.93 | 59.85±12.26 | 46.81±8.33 | 52.30±10.64 | 55.93±10.24 |
| 61-70/0-9 | 58.00±13.01 | 80.93±7.97 | 22.56±4.89 | 65.63±14.18 | 58.15±10.01 |

Data is presented as mean±SD unless mentioned otherwise. NCT: Number connection test; DST: Digit symbol test; LTT: Line tracing test; SDT: Serial dotting test.

**Supplementary Table 2. Description of metabolic pathway**

| **Control-MHE** | | |
| --- | --- | --- |
| **pathway code** | **Metabolic pathway description** | **q-value** |
| PWY-5532 | nucleoside and nucleotide degradation (archaea) | 0.003 |
| THREOCAT-PWY | superpathway of L-threonine metabolism | 0.003 |
| PWY-7013 | (S)-propane-1,2-diol degradation | 0.003 |
| PWY-6641 | superpathway of sulfolactate degradation | 0.018 |
| HEXITOLDEGSUPER-PWY | superpathway of hexitol degradation (bacteria) | 0.018 |
| PWY-1541 | superpathway of taurine degradation | 0.018 |
| LPSSYN-PWY | superpathway of Kdo2-lipid A biosynthesis | 0.018 |
| PWY-5920 | superpathway of heme b biosynthesis from glycine | 0.023 |
| PWY-1861 | formaldehyde assimilation II (assimilatory RuMP Cycle) | 0.030 |
| RUMP-PWY | formaldehyde oxidation I | 0.030 |
| PWY-5431 | aromatic compounds degradation via β-ketoadipate | 0.030 |
| PWY-5417 | catechol degradation III (ortho-cleavage pathway) | 0.030 |
| PROTOCATECHUATE-ORTHO-CLEAVAGE-PWY | protocatechuate degradation II (ortho-cleavage pathway) | 0.031 |
| PWY-5181 | toluene degradation III (aerobic) (via p-cresol) | 0.031 |
| CATECHOL-ORTHO-CLEAVAGE-PWY | catechol degradation to β-ketoadipate | 0.031 |
| PWY-6182 | superpathway of salicylate degradation | 0.033 |
| 3-HYDROXYPHENYLACETATE-DEGRADATION-PWY | 4-hydroxyphenylacetate degradation | 0.036 |
| PWY-6185 | 4-methylcatechol degradation (ortho cleavage) | 0.036 |
| GLYCOCAT-PWY | glycogen degradation I | 0.036 |
| METH-ACETATE-PWY | methanogenesis from acetate | 0.036 |
| GALLATE-DEGRADATION-II-PWY | gallate degradation I | 0.036 |
| PWY-5677 | succinate fermentation to butanoate | 0.037 |
| PWY0-1277 | 3-phenylpropanoate and 3-(3-hydroxyphenyl)propanoate degradation | 0.049 |
| PWY-5507 | adenosylcobalamin biosynthesis I (anaerobic) | 0.05 |
| HCAMHPDEG-PWY | 3-phenylpropanoate and 3-(3-hydroxyphenyl)propanoate degradation to 2-hydroxypentadienoate | 0.05 |
| PWY-6690 | cinnamate and 3-hydroxycinnamate degradation to 2-hydroxypentadienoate | 0.05 |
| **Pro-MHE** | | |
| **pathway code** | **Metabolic pathway description** | **p-value** |
| LACTOSECAT-PWY | lactose and galactose degradation I | 3.46E-03 |
| PWY-5265 | peptidoglycan biosynthesis II (staphylococci) | 4.69E-03 |
| 3-HYDROXYPHENYLACETATE-DEGRADATION-PWY | 4-hydroxyphenylacetate degradation | 8.19E-03 |
| PWY-1541 | superpathway of taurine degradation | 9.57E-03 |
| PWY-6470 | peptidoglycan biosynthesis V (β-lactam resistance) | 0.011 |
| PWY-3661 | glycine betaine degradation I | 0.012 |
| PWY-5941 | glycogen degradation II | 0.015 |
| PWY-6641 | superpathway of sulfolactate degradation | 0.015 |
| PWY-6562 | norspermidine biosynthesis | 0.016 |
| P562-PWY | *myo*-inositol degradation I | 0.017 |
| PWY-6071 | superpathway of phenylethylamine degradation | 0.021 |
| PWY0-321 | phenylacetate degradation I (aerobic) | 0.021 |
| METHYLGALLATE-DEGRADATION-PWY | methylgallate degradation | 0.022 |
| GALLATE-DEGRADATION-I-PWY | gallate degradation II | 0.022 |
| PWY-5431 | aromatic compounds degradation via β-ketoadipate | 0.023 |
| PWY-5417 | catechol degradation III (ortho-cleavage pathway) | 0.023 |
| GALLATE-DEGRADATION-II-PWY | gallate degradation I | 0.024 |
| PWY-6876 | isopropanol biosynthesis (engineered) | 0.027 |
| CATECHOL-ORTHO-CLEAVAGE-PWY | catechol degradation to β-ketoadipate | 0.031 |
| PWY0-41 | allantoin degradation IV (anaerobic) | 0.041 |
| **Rif-MHE** | | |
| **pathway code** | **Metabolic pathway description** | **q-value** |
| PWY-2941 | L-lysine biosynthesis II | 7.87E-03 |
| PWY-6470 | peptidoglycan biosynthesis V (β-lactam resistance) | 7.87E-03 |
| LACTOSECAT-PWY | lactose and galactose degradation I | 8.14E-03 |
| **Lac-MHE** | | |
| **pathway code** | **Metabolic pathway description** | **q-value** |
| ENTBACSYN-PWY | enterobactin biosynthesis | 0.020 |
| AST-PWY | L-arginine degradation II (AST pathway) | 0.020 |
| ORNARGDEG-PWY | superpathway of L-arginine and L-ornithine degradation | 0.022 |
| ARGDEG-PWY | superpathway of L-arginine, putrescine, and 4-aminobutanoate degradation | 0.022 |
| PWY0-1338 | polymyxin resistance | 0.022 |
| FAO-PWY | fatty acid β-oxidation I (generic) | 0.024 |
| ECASYN-PWY | enterobacterial common antigen biosynthesis | 0.025 |
| CODH-PWY | reductive acetyl coenzyme A pathway I (homoacetogenic bacteria) | 0.033 |
| PWY-6641 | superpathway of sulfolactate degradation | 0.033 |
| PWY0-1415 | superpathway of heme b biosynthesis from uroporphyrinogen-III | 0.033 |
| 3-HYDROXYPHENYLACETATE-DEGRADATION-PWY | 4-hydroxyphenylacetate degradation | 0.033 |
| PWY-1541 | superpathway of taurine degradation | 0.033 |
| TCA-GLYOX-BYPASS | superpathway of glyoxylate bypass and TCA | 0.041 |
| REDCITCYC | TCA cycle VI (Helicobacter) | 0.041 |
| ORNDEG-PWY | superpathway of ornithine degradation | 0.043 |
